# Quality of Primary Health Care for children and adolescents living with HIV**^1^**

**DOI:** 10.1590/1518-8345.0609.2720

**Published:** 2016-08-29

**Authors:** Leticia do Nascimento, Cristiane Cardoso de Paula, Tania Solange Bosi de Souza Magnago, Stela Maris de Mello Padoin, Erno Harzheim, Clarissa Bohrer da Silva

**Affiliations:** 2 RN, Hospital Universitário de Santa Maria, Santa Maria, RS, Brazil.; 3 PhD, Adjunct Professor, Centro de Ciências da Saúde, Universidade Federal de Santa Maria, Santa Maria, RS, Brazil.; 4 PhD, Adjunct Professor, Universidade Federal do Rio Grande do Sul, Porto Alegre, RS, Brazil.; 5 MSc, Assistant Professor, Faculdades Integradas de Taquara, Taquara, RS, Brazil.

**Keywords:** HIV, Acquired Immunodeficiency Syndrome, Primary Health Care, Evaluation of Health Services

## Abstract

**Objective:**

to evaluate the quality of health care for children and adolescents living with HIV, among the different types of Primary Health Care services of Santa Maria, Rio Grande do Sul.

**Method:**

cross-sectional study, developed with 118 Primary Health Care professionals. The Primary Care Evaluation Instrument, Professional version, was used. For verification of the variables associated with the high score, Poisson Regression was used.

**Results:**

the professionals of the Family Health Strategy, when compared to those of the Primary Health Units, obtained a greater degree of orientation to primary care, both for the overall score and for the derived attributes score, as well as for the integrality and community orientation attributes. A specialization in Primary Health Care, other employment and a statutory work contract were associated with quality of care.

**Conclusion:**

the Family Health Strategy was shown to provide higher quality health care for children and adolescents living with HIV, however, the coverage is still low. The need was highlighted to expand this coverage and invest in vocational training directed toward Primary Care and making the professionals effective, through public selection procedure, as well as an improvement program that recognizes the care requirements, in these settings, of children and adolescents infected with HIV.

## Introduction

Children and adolescents living with the Human Immunodeficiency Virus (HIV) or Acquired Immune Deficiency Syndrome (AIDS) present specific requirements due to their serologic status. They need constant monitoring in the healthcare services, for prevention of disease and maintenance of health^1^. Currently, this care mainly takes place in reference services due to the organization of specific services and skills of the professionals^2^ of the specialized services.

While there is a growing epidemiological representation of chronic conditions in Brazil, among them HIV, historically, the Primary Health Care (PHC) services have been geared to meet acute problems, negatively affecting the efficiency and quality of permanent health monitoring. Fragmentation in the organization of these services may impair the coordination of care by the PHC^3^. Strengthening PHC is a strategy to qualify the care for vulnerable groups in the Brazilian National Health System , coordinating the flow of patients in the care networks and increasing communication between the specialist services and the PHC^2^ ^-^ ^3^.

Primary Health Care can be defined as a set of values, principles and structural and complementary attributes of the health system. Its effectiveness is through means of its essential attributes (first contact, longitudinality, integrality and coordination of the care) and derived attributes (family-centered care and community orientation)^4^. The evaluation of the presence and extent of these attributes supports the (re)definition of public policies^5^.

For the evaluation of PHC quality the Primary Care Assessment Tool-Brazil (PCATool-Brazil) can be highlighted, which measures the presence and extent of attributes, with an emphasis on aspects of the structure and process of the services^4^ ^-^ ^5^. It is applicable as child, adult and professional versions, used nationally^6^ ^-^ ^8^ and internationally^9^ ^-^ ^11^ and includes chronic^12^, infectious and communicable conditions^13^ ^-^ ^15^. The gap in knowledge production with the population living with HIV is highlighted. The development of this study is justified by the need to evaluate the performance and structure of the PHC as the preferred point of entry into the Brazilian health system for children and adolescents living with HIV, in order to analyze the care network possibilities, to expand the actions developed in the specialized service.

The aim of this study was to evaluate the quality of healthcare for children and adolescents living with HIV, among the different types of PHC services in Santa Maria, Rio Grande do Sul (RS), Brazil, through the Professional version of the PCATool- Brazil.

## Method

This cross-sectional study was developed in the PHC services network of Santa Maria, RS. In this municipality, the public PHC network is made up of different types of services, under the responsibility of the Municipal Health Department, which are: Primary Health Units (PHUs), which contains the Community Health Agents Strategy (CHAS), and the Family Health Strategy (FHS). There are 31 PHC services, of which 18 are PHUs and 13 FHS.

The study population consisted of all health professionals that met the following inclusion criteria: physicians, nurses and dentists working in the PHC of Santa Maria, RS. Those professionals on vacation, sick leave or away from work during the period of data collection were excluded. The population totaled 122 professionals, of which two (1.64%) did not meet the inclusion criteria. Of the eligible population, 120 professionals, there were two (1.64%) refusals. The study population was 118 health professionals, with 89 from PHUs and 29 FHS.

For the characterization of the professionals, an instrument was used with sociodemographic variables regarding academic formation and occupational status (independent variables). The evaluation of quality of the PHC (dependent variable), in this study, was considered as presence and extent of the essential and derived attributes^4^. The Professional version of the PCATool-Brazil, consisting of 77 items, divided into six attributes, was applied in interview format. The responses are given on a Likert scale, being "certainly yes" (value=4), "probably yes" (value=3), "probably no" (value=2), "certainly no" (value=1) and "do not know/do not remember" (value=9). The responses marked "do not know/do not remember" were considered "probably no"^16^.

Data collection took place from January to July 2013, by previously trained research assistants. The interviews with the professionals were performed in a room that guaranteed privacy, in the service where they worked, during work time. They were instructed to respond to the instrument focusing on the care of children and/or adolescents living with HIV. The average time taken to complete the instrument was 40 minutes.

For storage of the data the Epi-Info(r), version 6.04 program was used, with independent double entry, to ensure data accuracy. After checking for errors and inconsistencies, the analysis was performed using the Predictive Analytics SoftWare (PASW), version 18.0 program for Windows.

The analysis of the PCATool reliability was performed using Cronbach's α (values>0.70 were considered indicators of consistency). The normal distribution of the variables was evaluated using the Kolmogorov-Smirnov test. The categorical variables were presented as absolute and relative frequency, and the continuous variables as mean and standard deviation, when presenting symmetrical distribution, and as median and interquartile range when asymmetrical.

For the characterization, the following sociodemographic variables were analyzed: gender (female, male), age (in years, dichotomous), academic formation variables: graduation, time since graduation (years, dichotomous), post-graduation (PHC and others), and occupational status variables: work unit (FHS and PHU), contract (CLT, statutory and outsourced, statutory and other), length of service (years, dichotomous), shift (morning, afternoon and mixed; single and mixed shift), other job (yes or no), managerial role in this service (yes or no).

For analysis of PCATool-Brazil, first the scores of the PHC attributes were calculated for all professionals. The values ​​that originally varied in the scale from 1 to 4 were processed on a continuous scale from 0 to 10. The scores were calculated for the essential attributes, derivatives, and the general PHC score (essential+derived), obtained by the arithmetic mean of the items that compose them. For a high score evaluation, values ​​≥6.6 were used, defined as the appropriate (satisfactory) extension of each attribute, this being values equivalent to 3 or over in the original Likert scale^16^.

Pearson's chi-square test with correction, when necessary, was used to compare proportions. To compare the mean scores of the attributes, depending on the type of unit (FHS and PHU), the Mann-Whitney U and Student's t tests were used. For all statistical analyzes, the significance level of 5% was adopted.

For verification of the variables associated with the high score, the Poisson regression with robust variance was used, with the prevalence ratios (PR) and their respective confidence intervals (95%CI) being estimated. The independent variables associated with the high score, with p<0.25, were included in the crude and adjusted analyzes.

The project was approved by the Research Ethics Committee of the Federal University of Santa Maria (UFSM) (CAAE: 12223312.3.0000. 5346) on 08/01/2013, respecting the current Resolution No. 196/96.

Results

Of the 122 professionals working in the PHC of Santa Maria, 118 (96.7%) participated. The losses (n=04; 3.3%) were due to refusal to participate. Table 1 shows the sociodemographic and employment characteristics of the PHC professionals, according to the evaluation of high and low PHC score.

**Table 1:** Distribution of the professionals, according to sociodemographic and employment characteristics and score for Primary Health Care. Santa Maria, RS, Brazil, 2013 (N=118)

Variable	PCATool-Brazil*				p	
	Low score (<6.6)	High score (≥6.6)				
	N	%	N	%		
Gender					0,590†
Male	19	44,2	24	55,8	
Female	37	49,3	38	50,7	
Age					0,975†
≤47 years	25	47,2	28	52,8	
>47 years	30	46,9	34	53,1	
Work unit					0,014†
Primary Health Unit	48	53,9	41	46,1	
Family Health Strategy	8	27,6	21	72,4	
Service contract (N=117)					0,009‡
Statutory	46	43,4	60	56,6	
CLT and outsourced	10	83,3	2	16,7	
Length of service					0,277†
≤7 years	26	42,6	35	57,4	
>7 years	30	52,6	27	47,4	
Shift					0,127†
Morning	24	60,0	16	40,0	
Afternoon	8	47,1	9	52,9	
Mixed	24	39,3	37	60,7	
Other job					0,074‡
No	29	56,9	22	43,1	
Yes	27	40,3	40	59,7	
Managerial role in this service					0,603†
No	42	46,2	49	53,8	
Yes	14	51,9	13	48,1	
Which managerial role (N=26)					0,289‡
Responsible technician of the service	10	62,5	6	37,5	
Coordinator of the service	2	28,6	5	71,4	
Responsible for community health agents	2	66,7	1	33,3	

*Primary Care Assessment Tool; †Pearson's chi-square test; ‡chi-square test with Monte Carlo correction

Regarding the PHC scores in Table 1, a statistically significant difference was shown among the results, in that the FHS professionals presented a higher percentage (72.4%; p=0.014) for the high score compared to those of the PHUs.

The formation profile variables of the professionals with the evaluation of high and low score for PHC can be seen in Table 2.

**Table 2:** formation profile of the professionals, according to the evaluation of high and low scores for Primary Health Care. Santa Maria, RS, Brazil, 2013 (N=118)

		PCATool-Brazil*			p
Variable	Low score (<6.6)			High score (≥6.6)	
	N	%	N	%	
Formation					0.600†
General surgery	13	38.2	21	61.8	
Gynecologist	8	53.3	7	46.7	
Pediatrician	4	36.4	7	63.6	
Nurse	18	52.9	16	47.1	
Dentist	13	54.2	11	45.8	
Time since graduation (N=117)					0.778†
≤4 years	29	49.2	30	50.8	
>24 years	27	46.6	31	53.4	
Post-graduation					0.378†
Do not have	11	61.1	7	38.9	
Residency	16	39.0	25	61.0	
Specialization	26	51.0	25	49.0	
MSc.	3	37.5	5	62.5	
Time since post-graduation					0.519†
≤9 years	22	44.0	28	56.0	
>9 years	34	50.0	34	50.0	
Post-graduation					0.378‡
Do not have	11	61.1	7	38.9	
Residency	16	39.0	25	61.0	
Specialization	26	51.0	25	49.0	
MSc.	3	37.5	5	62.5	
Residency (N=14)					0.375‡
Collective health/family medicine	1	20.0	4	80.0	
Others^§^	15	41.7	21	58.3	
Specializations^||^ (N=63)					0.076‡
Community/collective/family/public health	17	40.5	25	59.5	
Others	14	66.7	7	33.3	
MSc (N=8)					0.804‡
Endodontics	-	-	1	100	
Geomatics	1	50.0	1	50.0	
Nursing	1	25.0	3	75.0	
Production engineering	1	100	-	-	
Additional training					0.809†
No	9	45.0	11	55.0	
Yes	47	48.0	51	52.0	

*Primary Care Assessment Tool; †Pearson's chi-square test; ‡chi-square test with Monte Carlo correction

§general surgery, urology, internal medicine, gynecology and obstetrics, pediatrics, gastroenterology, psychiatry

||some professionals had completed more than one specialization

When assessing the formation characteristics related to the PHC scores, there were no statistical differences between the groups.

Table 3 presents the descriptive statistics of the PHC attributes, measured from the experience of the professionals with healthcare of children and adolescents living with HIV.

**Table 3:** Descriptive statistics of the attributes of Primary Health Care, measured from the perception of the professionals regarding the healthcare of children and adolescents living with HIV. Santa Maria, RS, Brazil, 2013

Attributes of Primary Health Care		Mean	Standard deviation	Median	Cronbach's alpha
Essential attributes					
	First-contact access*	4.02	1.30	3.70	0.43
	Longitudinality*	6.70	1.37	6.41	0.73
	Integrality- services available†	6.13	1.54	6.28	0.69
	Integrality- services provided*	6.45	3.04	7.33	0.86
	Coordination - care integration*	6.97	1.56	6.94	0.56
	Coordination - information system*	8.44	1.61	8.89	0.18
Derived attributes					
	Family orientation*	7.80	2.36	8.89	0.74
	Community orientation*	5.41	2.03	5.56	0.69
General evaluation					
	Score of the essential attributes†	6.45	1.06	6.63	0.83
	Score of derived attributes*	6.61	1.88	6.94	0.77
	General score*	6.49	1.15	6.69	0.87

*normal distribution; †asymmetric distribution

In the general assessment of the PHC of the municipality, it can be observed that, of the six essential dimensions, three presented satisfactory assessments (mean ≥6,6): longitudinality of the care (mean=6.7), coordination- care integration (mean=6.97) and coordination- information system (mean=8.44). Of the two derived attributes, only family orientation was above the cut-off point (mean=7.80).

The general mean of derived attributes presented a value above that indicated (mean=6.61), considered appropriate extension. The internal consistency of the instrument (α=0.87) and its essential (α=0.83) and derived (α=0.77) attributes was adequate.

Table 4 shows the essential and derived attribute scores and the general scores of the PHC, for both types of services evaluated.

**Table 4:** Comparison of the scores of the Primary Health Care attributes in relation to the health care of children and adolescents living with HIV, given by professionals. Santa Maria, RS, Brazil, 2013 (N=118)

		Scores (0-10)				p		
					Family Health Strategy (n=29)			
		Mean	Standard deviation	Median	Mean	Standard deviation	Median	
Essential attribute								
	Access	4.05	1.45	3.70	3.93	0.64	3.70	0.890*
	Longitudinality	6.58	1.39	6.41	7.06	1.26	6.67	0.199*
	Integrality (available services)	6.10	1.56	6.15	6.22	1.52	6.41	0.726†
	Integrality (services provided)	5.89	3.21	6.67	8.16	1.49	8.00	0.002*
	Coordination (care integration)	7.00	1.57	7.22	6.88	1.54	6.67	0.758*
	Coordination (information system)	8.3	1.72	8.89	8.85	1.21	8.89	0.158*
Derived attributes								
	Family orientation	7.58	2.54	7.78	8.47	1.55	8.89	0.183*
	Community orientation	4.98	2.01	5.00	6.76	1.42	6.67	<0.001*
General evaluation								
	Essential score	6.32	1.12	6.53	6.85	0.73	6.78	0.201†
	Derived score	6.28	1.93	6.67	7.61	0.13	7.78	0.001*
	General score	6.30	1.21	6.48	7.04	0.72	7.06	0.003*

*Mann Whitney test; †Student's t-test

In the evaluation of the PHC attributes, according to the type of unit, the FHS professionals scored significantly higher means in the integrality (services provided) (8.16 vs. 5.89) and community orientation (6.76 vs. 4.98) items, compared to those of the PHUs. The same was the case for the derived attribute scores (7.61 vs. 6.28) and general score (7.04 vs. 6.30) of the PHC.

Table 5 presents the crude and adjusted analyzes for the general score of the PHC of Santa Maria, attributed by the professionals to the healthcare of children and adolescents living with HIV.

**Table 5:** Gross and adjusted regression for the general score of the Primary Health Care attributed by the professionals to the healthcare of children and adolescents living with HIV. Santa Maria, RS, Brazil, 2013

Variables		High score					p
		GPr *	95%CI^†^	p	APr^‡^	95%CI^†^	
Have specialization							
	Primary healthcare	1.08	0.96-1.22	0.182	1.02	0.90-1.15	0.747
	Others	1.00			1.00		
Have another job							
	Yes	1.12	0.99-1.26	0.074	1.19	1.06-1.33	0.002
	No	1.00			1.00		
Type of service							
	Family Health Strategy	1.18	1.05-1.33	0.006	1.21	1.04-1.14	0.012
	Primary Health Unit	1.00			1.00		
Service contract							
	Statutory	1.34	1.11-1.62	0.002	1.37	1.20-1.57	<0.00
	Others	1.00			1.00		1
Shift							
	Mixed	1.12	0.99-1.25	0.66	1.05	0.91-1.20	0.492
	Morning or afternoon	1.00			1.00		

GPr* Gross Poisson regression

†95%CI: 95% confidence interval

‡APr: Poisson regression adjusted for: type of service, specialization, contract, shift and other job

After the adjustments, the following were associated with the high score: having another job, working in the FHS and having a statutory contract.

## Discussion

The FHS professionals presented higher percentages of high scores for the PHC than those linked to the PHUs, corroborating comparative studies^8^ ^,^ ^17^. The general assessment of the PHC was satisfactory for the longitudinality of the care, coordination - care integration, coordination - information system and family orientation attributes. This is in agreement with studies performed in East Asia, which showed that primary care presents higher performance in these attributes^10^ ^-^ ^11^.

As was found by a study carried out in Porto Alegre, RS^18^, the values ​​assigned to the general and essential attribute scores of the PHC of Santa Maria, RS, were considered unsatisfactory. However, the mean general score of the derived attributes was considered appropriate extension, as in the East Asian studies that found a higher mean for this^10^ ^-^ ^11^.

When comparing the types of service, statistically significant difference was evidenced between the means of the general score, highlighting that the FHS presents a higher degree of orientation toward PHC (7.04 vs. 6.30). A study conducted in Porto Alegre, RS, also showed a significant difference in favor of the FHS (7.08 vs. 6.58)^17^. A statistically significant difference was found for the derived attributes score, convergent with the characteristics inherent to the FHS (7.61 FHS vs. 6.28 PHUs). This result is in agreement with comparative studies, which showed significant differences in favor of the FHS in the derived attributes^6^ ^,^ ^8^ ^-^ ^17^.

The attributes integrality - services provided (8.16 vs. 5.89) and community orientation (6.76 vs. 4.98) stand out, with statistical significance. This is in agreement with the assessment carried out in Porto Alegre, RS, which found statistically significant higher scores in the integrality- services provided (8.27 vs. 7.02) and community orientation (6.75 vs. 5.58) attributes^17^. In a study conducted in the USA, which focused on chronic conditions, improved continuity of the treatment was demonstrated among patients who received care and guidance in their homes^12^. Studies highlight the need for improvements in the actions of social partnerships, in the active search and in community participation regarding infectious and contagious diseases^13^ ^-^ ^15^.

The analysis of the integrality of the services available did not distinguish the FHS from the PHUs, with both presenting scores below the ideal, unlike studies in which professionals of the FHS gave this attribute a positive evaluation^6^ ^,^ ^17^. However, a study revealed that, regardless of the use of the PHC services, people living with HIV preferably seek specialized services, as they consider them capable of ensuring integrality in the healthcare, negatively interfering with the service network^2^.

The first contact access was configured as the worst performers, in both the FHS and the PHUs. This finding is in agreement with other studies that indicated this to be a bottleneck of the system^7^ ^-^ ^8^. These difficulties of access have an influence on organizational issues of the services, such as the organization of the schedule, difficulty making appointments and care programs, among others. This can influence the PHC as the point of entry^19^. A study performed in João Pessoa, PB, with caregivers of children with chronic diseases, confirmed that the PHC is not qualified as the point of entry of the system and showed it to be weak in meeting the demands in resolutive way, contributing to services of medium and high technological density being sought^20^. However, it should be noted that a study conducted in the municipality on the border of Brazil, Paraguay and Argentina, revealed that the patients were diagnosed later when the PHC was the service of first choice for treatment of infectious and contagious diseases, which highlights the need for investment in the quality of PHC^21^.

The coordination of the care presented a satisfactory evaluation in both types of service, which is in agreement with a study that considered this feature to be present in PHC in general^8^ ^,^ ^10^, without highlighting the FHS. The satisfactory evaluation demonstrated for this attribute, in both services, may be due to the perception of the need to combine the primary care network and specialized services for the health of the population living with HIV^2^ ^-^ ^3^.

The evaluation of longitudinality was satisfactory in the FHS (p>0.05). A study, conducted in the states of Goiás and Mato Grosso do Sul, indicated that the FHS presented a significant difference in the bond with users when compared to PHUs^8^. This bond is a result of confidence in the professional, favoring the resolution and referral of health problems, reducing the need to use services of high technological density^22^. However, a study carried out in Ribeirão Preto, SP, with users living with HIV, showed the need to improve the communication and acceptance of professionals, this being essential for the process of adherence and continuity of the health monitoring^2^.

The assessment of family orientation was satisfactory in both services (p>0.05), as in studies involving PHC in Brazil, the United States and Taiwan^8^ ^-^ ^9^ ^,^ ^11^ ^,^ ^17^ ^,^ ^23^. A study performed with family members found that social, cognitive psychological and resource support favor the improvement of family well-being, confirming the importance of care directed toward the family^24^.

Adjusted multivariate analysis confirmed a higher prevalence in the high PHC score, when professionals have another job, work in the FHS and have a statutory contract. The high number of statutory professional is a particular feature of the municipality studied, which is positively in favor of PHC. In many cities of the South of Brazil, the staff turnover causes an impasse in the implementation of the FHS^25^. Finally, having another job appeared as a novel finding and may be associated with the comparison that the professionals made between the different services in which they participate.

The importance of studies of PHC assessment is emphasized, considering the limits of this study: the instrument is not specific to people with HIV, preventing the evaluation of particularities; the results are limited to the characteristics of a single municipality; and there is a possible bias of reverse causality common to cross-sectional studies, which do not guarantee temporality. This study contributes to the advancement of scientific knowledge in that it reiterates the qualification of healthcare in the presence of the FHS, which indicates the expansion of the coverage of this type of service. It also indicates the importance of evaluating the quality of healthcare and the need for investment in instruments dealing with the specificity of the population living with HIV who are mostly affiliated with specialized service, considering the perspective of care in the healthcare network.

## Conclusion

Regarding the service models in PHC, the FHS stands out with the highest degree of orientation toward PHC. However, the FHS coverage in the city is still low (21%), which means that the largest proportion of the population receives health care with a lower degree of orientation, highlighting the need to expand the FHS coverage.

By identifying the variables associated with the high PHC score, having another job, working in the FHS, and having a statutory contract, shows that investment in the qualification of the professional directed toward PHC, as well as the expansion of coverage of the FHS and making the professionals effective, through public selection procedure, are strategies for qualifying the PHC. The need is emphasized for inclusion, in PHC, of a training program for professionals that recognizes the care requirements of the population of children and adolescents living with HIV in this scenario.
